# Discriminating Gene Expression Signature of Radiation-Induced Thyroid Tumors after Either External Exposure or Internal Contamination

**DOI:** 10.3390/genes3010019

**Published:** 2011-12-21

**Authors:** Catherine Ory, Nicolas Ugolin, Martin Schlumberger, Paul Hofman, Sylvie Chevillard

**Affiliations:** 1 CEA, DSV, IRCM, SREIT, Laboratoire de Cancérologie Expérimentale, BP6, Fontenay-aux-Roses, F-92265, France; E-Mails: nicolas.ugolin@cea.fr (N.U.); sylvie.chevillard@cea.fr (S.C.); 2 Institut Gustave Roussy, Department on Nuclear Medicine and Endocrine Oncology, Villejuif, and University Paris-Sud, F-94800, France; E-Mail: Martin.SCHLUMBERGER@igr.fr; 3 INSERM ERI-21, Nice, F-06002, France; E-Mail: HOFMAN.P@chu-nice.fr; 4 University of Nice-Sophia Antipolis, IFR 50, Nice, F-06002, France; 5 Laboratory of Clinical and Experimental Pathology and CHU-CRLCC-UNSA tumour tissue bank of Nice area, Louis Pasteur Hospital, Nice, F-06002, France

**Keywords:** thyroid, radiation-induced tumors, transcriptomic signature, molecular epidemiology

## Abstract

Both external radiation exposure and internal radionuclide contamination are well known risk factors in the development of thyroid epithelial tumors. The identification of specific molecular markers deregulated in radiation-induced thyroid tumors is important for the etiological diagnosis since neither histological features nor genetic alterations can discriminate between sporadic and radiation-induced tumors. Identification of highly discriminating markers in radiation-induced tumors is challenging as it relies on the ability to identify marker deregulation which is associated with a cellular stress that occurred many years before in the thyroid cells. The existence of such a signature is still controversial, as it was not found in several studies while a highly discriminating signature was found in both post-radiotherapy and post-Chernobyl series in other studies. Overall, published studies searching for radiation-induced thyroid tumor specificities, using transcriptomic, proteomic and comparative genomic hybridization approaches, and bearing in mind the analytical constraints required to analyze such small series of tumors, suggest that such a molecular signature could be found. In comparison with sporadic tumors, we highlight molecular similarities and specificities in tumors occurring after high-dose external radiation exposure, such as radiotherapy, and in post-Chernobyl tumors that occurred after internal ^131^I contamination. We discuss the relevance of signature extrapolation from series of tumors developing after high and low doses in the identification of tumors induced at very low doses of radiation.

## 1. Radiosensitivity of Thyroid Tissue in Childhood

Radiation exposure during childhood is a well demonstrated pro-tumorigenic factor for thyroid tissues. Increased risk of developing a thyroid tumor has been reported in epidemiologic studies on cohorts of children or young adults externally exposed during radiotherapy for benign diseases or for a primary cancer [1,2,3] and after the atomic bombing of Japan [4] or internally contaminated by ^131^I after Chernobyl fallout [5,6]. Thyroid tissue radiosensitivity in adults is much lower than for children, if it exists [7,8]. The reported excess relative risk of thyroid cancer exposure is at its highest when exposure occurs in early childhood and decreases with exposure at older age; some other factors, such as gender or iodine supply, may modify the risk [7,8]. It was an estimated 1.31 per Gray (Gy) in a study analyzing a cohort of childhood cancer survivors [2], 1.91 per Gray (Gy) in a recent study analyzing a post-Chernobyl Ukrainian cohort [9], 7.7 per Gy in a meta-analysis of several studies of externally exposed cohorts [10], and even higher in Hiroshima-Nagasaki cohorts. The risk is significant for doses as low as 0.1 Gy [10], increases linearly with doses up to 15–20 Gy [2], and then decreases because of a cell-killing effect at higher doses. Analysis of the Israël tinea capitis cohort, covering a dose range below 0.1 Gy, by Ron *et al.* and Sadetzki *et al*.; reported an ERR per Gy of 32.5 and 20.2, respectively [10,11]. However, estimation of the risk measured with this series is much higher than any other series and may not be extrapolated to others series since unique genetic background is suspected [11]. The risk peaks 15 years after exposure but remains significant over decades, more than 40 years in the study by Ron *et al.* [10]. Accordingly, an excess of thyroid cancer is still being observed post-Chernobyl, mainly in people exposed at younger ages [9].

## 2. Do Radiation-Induced Thyroid Tumors Show Specific Clinical Features?

Radiation-induced thyroid cancers are mainly papillary thyroid carcinomas (PTCs) which are also predominantly found in sporadic tumors. PTCs are found almost exclusively among short latency post-Chernobyl tumors, and include a high percentage of solid PTC variants, but this is related more to the association of the solid PTC variant with younger age of diagnosis of the tumor rather than with the radiation exposure [12,13]. The effect of low-iodine diet has also been reported as a risk factor for the development of these thyroid tumors, both by increasing the uptake of radioiodine in the thyroid and therefore the dose of radiation delivered to the thyroid gland and by promoting thyroid cancer growth [14,15]. Follicular adenomas and a few follicular carcinomas are also found in cohorts exposed to radiation [12,15,16,17,18].

An increased frequency of RET/PTC rearrangement over BRAF mutations is associated with radiation-induced DNA breakdown, but such an increase is also found in the absence of radiation exposure in young patients as compared with adult tumors. A disequilibrium of RET/PTC3 over RET/PTC1 has been described, and is associated with the short latency solid variant of PTC. Increased aggressiveness of radiation-induced tumors is also a feature associated with short latency pediatric tumors, including solid PTC variants [14,15,19,20], indicating that age at diagnosis more than radiation exposure is the parameter correlated with aggressiveness [19]. The nature and the frequency of molecular alterations found in radiation-induced tumors are associated with many parameters (age at radiation exposure, radiation dose, latency for tumor occurrence or histological subtype or other factors) unrelated to radiation exposure [21,22,23,24,25,26,27]. Thus, no etiology imprinting is currently suspected in a given tumor regarding clinical data, histopathological features and molecular alterations.

## 3. High-Throughput Molecular Studies of Radiation-Induced Thyroid Tumors: Can They Identify Discriminating Radiation-Induced Markers?

Radiation-induced molecular specificities have been sought using transcriptome analysis [28,29,30,31,32,33,34], proteome analysis [35,36] and genomic analysis [32,37,38,39]. Few studies have been published to date and most have focused on post-Chernobyl tumors due to internal ^131^I contamination; only one study has reported the identification of a discriminating transcriptomic radiation-induced signature after external exposure (post-radiotherapy) [33].

In two of these studies, molecular specificities of post-Chernobyl tumors were sought but not found at the transcriptomic level [28,31], and the authors suggested that radiation-induced and sporadic thyroid tumors have very similar transcriptomic patterns. However, the other studies have found sets of deregulated genes/proteins that more or less accurately identified post-Chernobyl [29,30,34,35], or post-radiotherapy induced tumors [33] from sporadic tumors (Table 1). However, only one group went one step further, by testing the robustness of the signatures by blind identification of the etiology of an independent series of thyroid tumors [33,34].

To date, limited overlap exists between the genes found to be deregulated in these studies (Supplementary table) and this may be due to differences in microarray platforms, data analysis and normalization, false positive rate, methods of tumor clustering, small size of the series [40], and/or specificities of the series of tumors (Table 1). For example, in comparison with other series of tumors, Abend *et al*. studied the transcriptome and the proteome of post-Chernobyl PTCs of shorter latency and compared them with those of sporadic tumors diagnosed in older patients as compared with other series. As expected, their markers suggested a higher aggressiveness of radiation-induced compared to sporadic tumors [30,35]. Other possible explanations of this low overlap between deregulated genes in the literature stem from the fact that published studies have not explored or reported full microarray data. Published sets of genes are sometimes limited to subsets of the identified deregulated genes [30] or to genes deregulated in post-Chernobyl *versus* normal tissue by subtraction of the genes already deregulated in sporadic PTCs *versus* normal tissue, thus eliminating genes that could be deregulated in both cases but at different levels [32]. Moreover, some authors focused on molecular mechanisms related to DNA repair, radiation, H_2_O_2_ or stress cellular response, and removed from analysis all genes of the immune response [29,41], these are however one of the radiation-associated features in other studies [30,33].

Molecular specificities of radiation-induced tumors are also sustained by a recent genomic approach used to analyze age at diagnosis- and ethnicity-matched sporadic and post-Chernobyl pediatric PTCs and identified a radiation-induced specific gain on chromosome 7q11.22–11.23 [38]. This locus includes genes involved in DNA repair, response to DNA damage and cell adhesion, and these categories of genes were previously reported to be abnormally expressed in several transcriptomic studies [29,30,33,34].

All this data suggests that molecular markers for classifying radiation-induced and sporadic thyroid tumors do exist, but most authors have not fully explored their data. Moreover, not all the usual bioinformatic tools for transcriptomic analysis are fully appropriate for the analysis of small series of radiation-induced tumors [31,34].

## 4. Highly Discriminating Radiation-Induced Thyroid Tumor Signature: Is It a Question of Methodology?

Whatever the tumor type a transcriptomic signature for classifying tumors could be masked or spoiled by the background noise inherent in the microarray technique, because most levels of gene expression do not differ significantly from one group to another and because of the possible heterogeneity of gene expression within a given subgroup of tumors [42]. Moreover, confounding factors such as age at irradiation and diagnosis, histology, gender, and gene alterations could, as a function of the methodology, result in biased selection of genes. Finally, the validation of the signature, by testing if it is suitable to blindly discriminate tumors, necessitates keeping apart a group of tumors. Of course, all these limitations are even more crucial when working with rare tumors (small series) such as radiation-induced tumors (Table 1). It is therefore hard to find the balance between a number of tumors that is large enough to find the signature, while having also enough tumors for the statistical validation of the signature prediction. Consequently, well-known conventional microarray analysis methods, which were successfully used in large tumor series and/or in series displaying a limited intra-group heterogeneity, appeared unsuitable for small series of radiation-induced thyroid tumors. Specifically, unsupervised or supervised tumor classification, generalized partial least-square, random forest, linear kernel support vector machine, prediction analysis of microarray, significance analysis of microarrays, gene expression bar code, top-scoring pair(s) and a PCA-based method applied by us and others, have either identified signatures of poor robustness [29,34], or have failed to find any signature [28,31] in post-radiotherapy or post-Chernobyl series.

**Table 1:** Published studies analyzing molecular transcriptomic or proteomic specificities of radiation-induced thyroid cancers.

	**Study**	**Port *et al.*; 2007**	**Detours *et al.*; 2007**	**Stein *et al.*; 2010 1**	**Ugolin *et al.*; 2,3**	**Ory *et al.*; 2011 3**	**Boltze *et al.*; 2009**
	**Exposure**	Post-Chernobyl	Post-Chernobyl	Post-Chernobyl	Post-Chernobyl	Post-radiotherapy	Post-Chernobyl
**Radiation-induced**	**Tumor set**	11 PTC 6	12 PTC	10 PTC	Learning set: 6 PTC	Learning set: 7 rPTC, 7rFTA	86 PTC
		3 males, 8 females	4 males, 8 females	Half male and half female	3 males, 3 females	4 males, 10 females	40 males, 46 females
	**Age at IR**	/	1b–16 years(M = 8.6 years)	2 monthb–14 years (M = 6 years)	10b–16 years (M = 12.7 years)	3b–14 years (M = 8.6 years)	3b–23 years (M = 11.8 years)
	**Age at diagnosis**	15b–22 years (M = 18 years)	16b–33 years (M = 24 years)	14b–28 years (M = 20 years)	27b–33 years (M = 29.2 years)	20b–56 years (M = 35.1 years)	12b–28 years (M = 18.6 years)
	**Latency**	Up to 15 years after 1986	15b–17 years (M = 16 years)	14b–16 years (M = 13.6 years)	16b–17 years (M = 16.5 years)	11b–48 years (M = 26.5 years)	Up to 15 years after 1986 (mean = 6.8 years)
	**Dosimetry**	90% at 0.15-1Gy 4	/	/	/	12b–42.5 Gy (M = 14.1 Gy)	90% at 0.15-1Gy 4
	**Histology**	PTC	8 PTC, 3 FVPTC, 1 PTC	PTC	? 7	7 FTA; 6 PTC; 1 FVPTC	PTC
	**Mutations**	2 PTC1; PTC3 6	5 BRAF; 5 PTC	1 PTC; 1 PTC3	4 BRAF; 1 RET/PTC	1 BRAF; 1 RAS	
	**Others**	T2N0M0 to T4N1M1	/	/	/	5 with chemotherapy	
**Sporadic**	**Tumor set**	41 PTC	14 PTC 7	20 PTC from He *et al.* study 5	Learning set: 7 PTC	Learning set: 7 sPTC, 7sFTA	91 PTC
		19 males, 22 females	5 males, 9 females	8 males, 12 females	4 males, 3 females	5 males, 9 females	49 males, 42 females
	**Age at diagnosis**	15b–83years (M = 60 years)	29b–68 years (M = 47 years)	13b–65 years (M = 44.4 years)	29b–38 years (M = 34.6 years)	21b–63 years (M = 37.6 years)	15b–83 years (M = 50.1 years)
	**Histology**	PTC 6	9 PTC, 4 FVPTC, 1 tPTC	14 PTC; 5 FVPTC; 1 HCC7	/ 7	7 FTA; 5 PTC; 2 FVPTC	PTC
	**Mutations**	5 PTC1	5/14 BRAF; 3/14 RET/PTC	/	2 BRAF; 1 RET/PTC	4 BRAF; 2 RAS, 1 PTC1, 1 PTC3	
	**Others**	T1N0-1M0 (n = 26) to T3N1M0	/	/		None with chemotherapy	
	**Arrays**	Human genome survey microarray V2.0 (Applied Biosystems) (33,000 probes)	Human 1 cDNA Microarray slides (Agilent Technologies). (19,000 probes)	Affymetrix U133A Array (20,000 probes) (Stein *et al.*)	Dataset retrieved from GEO (GSE3950)	Human 25K 50b–52mer oligo-microarrays (national genomic platform)	(Not relevant)
			Hybridized with normal matched tissue	Affymetrix U133 Plus 2.0 Array (50,000 probes) (He *et al.*) Hybridized with normal matched tissue		Hybridized with an internal reference (pool of normal thyroid tissues)	
	**Analysis**	Identified 1300 genes up- or downregulated at least fivefold (pool of 10 rPTC *vs.* pool of 10 sPTC)	(1) Several methods applied for tumor classification	Compared two sets of deregulated genes obtained separately: (1) post-Chernobyl PTC *vs.* normal tissues and; (2) sporadic PTC *vs.* normal tissues	106 genes discriminating signature identified by applying the EMts_PCA on the learning/training set	322-gene discriminating signature identified by applying the EMts_PCA to the learning/training set	Identification of protein markers by MALDI-TOF mass spectrophotometry
		Validation of 92 more deregulated genes in the full tumor series by RT-PCR	(2) Same methods applied for tumor classification by using a γ-irradiation *vs.* H2O2 lymphocyte response signature.	Retained the genes deregulated in post-Chernobyl PTC only	651 deregulated genes identified	1900 deregulated genes identified	20 candidate protein markers analyzed by immunochemistry
	**Results**	10 genes for complete separation of the groups (no validation on an independent tumor sets)	In both cases classification with error rate errors of 8 to 42% for sporadic tumors and 7 to 29% for post-Chernobyl tumors	Identified 177 deregulated genes unique to the radiation-induced tumors	Etiology prediction of the 13 remaining tumors using the 106 gene signature (1 unclassified, non misclassified)	Blind prediction of etiology of the 29 remaining tumors (tumors (13 rPTC or FTA; 16 sPTC FTA) (1 unclassified, 2 misclassified)	Combination of 6 of these markers separates the groups (no validation on an independent tumor sets)

PTC: Papillary thyroid carcinoma; FVPTC: PTC, follicular variant, tPTC: PTC, trabecular variant; PTCs: PTC, solid variant; HCC: Hurthle cell carcinoma (HCC with follicular and papillary features); FTA: follicular thyroid adenoma; rPTC, rFTA: radiation-induced PTC; FTA; sPTC, sFTA: sporadic PTC; FTA; RAS: mutation in NRAS, HRAS or KRAS gene; BRAF: V600E BRAF mutation; PTC (unspecified), PTC1, PTC3: RET/PTC rearrangement; M: mean; ^1^ Transcriptome analysis was performed on 10 out 14 PTC tumors described in Stein *et al*. [32]. Clinical data are given for the full tumor set; ^2^ From Detours *et al*. [29]. For the analysis described in Ugolin *et al*. [34], 6 out of 12 post-Chernobyl PTCs and 7 out 14 sporadic PTCs of the tumor set described by Detours *et al*. [29], were used as a learning/training set for signature identification, the remaining tumors were used as testing set. Clinical data are given for the learning/training set; ^3^ Clinical data are given for the learning/training set; ^4^ Estimation from general dosimetry data; ^5^ From the He *et al*. study, 2005 (GSE3467); ^6^ No indication of the 10 tumors used for pool; ^7^ No indication of the precise histology by tumor.

To circumvent these limitations, we have proposed a new microarray analysis strategy, the EMts_2PCA method ([34], see supplementary data), which is specifically adapted to identify sets of genes with a high discriminating potential by using small series of samples (as small as 6–7 tumors in each sporadic and radiation-induced group) and which greatly limits the effect of confounding factors (sex, age at diagnosis, gene alteration, histology, TNM classification..) in the signature of interest, as the search for discriminating genes can be focused specifically on one criterion, for example the etiology. Robustness of the signature is then assessed by the blind validation on an independent set of tumors at least of the same size as the tumor set used to identify the signature [34]. This strategy was successfully used to find discriminating signatures in series of post-radiotherapy and post-Chernobyl thyroid tumors [33,34], but also in radiation-induced sarcomas [43], and in radiation-induced breast tumors by reanalyzing the published dataset of Broeks *et al.* [34,44].

Thus, by using a dedicated method, such as EMts_2PCA, focused on the identification of markers of high potential for etiology prediction and which greatly limits the effect of confounding factors, a radiation-induced highly discriminating signature was identified in both post-Chernobyl and post-radiotherapy thyroid tumors.

## 5. Are Specific Molecular Pathways Deregulated in Radiation-Induced Thyroid Tumors?

Despite the limited overlap in term of genes markers, analysis of the deregulated genes identified in radiation-induced tumors, in terms of functional categories or molecular pathways, give some preliminary indications about thyroid radiation-induced tumorigenesis. Upregulated genes identified by Port *et al*. are mainly associated with signal transduction (G proteins, VEGF-A, PDGF-B and EGF signaling pathways) and metabolic processes (oxidoreductases related to fatty acid desaturation, and steroid metabolism). Downregulated genes are mainly associated with the immune response, signal transduction (G protein and cytokines/chemokines) and cell communication/adhesion [30]. Immune response and signal transduction pathways including EGFR, MAPK, Rac/cdc42, hedgehog, TGF/BMP, calcium signaling and WNT canonical and noncanonical pathways are also deregulated in post-radiotherapy tumors [33]. Detours, *et al*. suggested that genes deregulated in post-Chernobyl tumors are mainly involved in cellular responses to γ-radiation and DNA double-strand break repair while sporadic tumors deregulated genes seem to bear the hallmark of the oxidative stress response [29]. However, analysis of the same series of tumors showed that post-Chernobyl tumors displayed a pattern of both DNA repair (including double-strand breaks, base and mismatch repair) and oxidative stress response [34]. The set of markers identified by Stein *et al*. [32] included predominant functional categories such as connective tissue development, cancer, cell cycle, gene expression, with a significant tendency to nucleic acid metabolism, the latter being also highly deregulated in the study by Ugolin *et al*. [34]. In the study of Stein *et al*., most of the post-Chernobyl deregulated genes were associated with an ESR1 and/or TERT focused network [32], while both ESR1 and ESR2 were found to be downregulated in post-radiotherapy tumors [33].

Interestingly, genes identified as participants in the response of the non-tumorigenic human thyroid epithelial cell line Htori-3 to high-linear energy transfer particles [45], in the human thyroid papillary carcinoma cell line TPC-1 to various doses of X-rays [46], or as participants in the specific response of human thyroid tissue transplanted in SCID mice to ^137^Cs or neutrons [47], are also found deregulated in radiation-induced thyroid tumors both in post-Chernobyl and post-radiotherapy series and may be markers of radiation exposure whatever the dose, dose rate, external radiation or internal contamination (Supplementary table).

## 6. Imprinting of Radiation-Induced Thyroid Tumors: Impact of the Mode of Exposure, Dose and Dose Rate

Both external exposure and contamination can induce thyroid tumors, but the relevance of extrapolation of conclusions from data on tumors occurring after exposure to external radiation, such as radiotherapy, to post-Chernobyl tumors that occurred after internal ^131^I contamination still has not been investigated. Chernobyl-exposed people received a median radiation dose of 0.37 Gy to the thyroid, and the dose ranged from less than 0.1 Gy to 10 Gy [48,49] while, in the case of thyroid tumors that develop after radiotherapy for a primary cancer during childhood, such as Hodgkin disease, the dose to the thyroid could easily be above the 15–20 Gy limit for observation of the cell-killing effect in thyroid tissue [2,33]. Thus, the impact of the dose to the thyroid may also be determinant for the selection of specific molecular mechanisms in the tumor in relation to the amount of DNA damage and the severity and nature of the cellular stress which the damaged thyrocytes will have to survive to lead to a tumor. In the context of the identification of a radiation-induced signature, the extent of similarity and specificities of molecular mechanisms in external exposure-induced and contamination-induced tumorigenesis and the impact of the dose are crucial as this should determine the possibility of identifying a general radiation-induced signature or should limit the use of mode of exposure- and dose range specific signatures for high robustness prediction.

**Figure 1:**
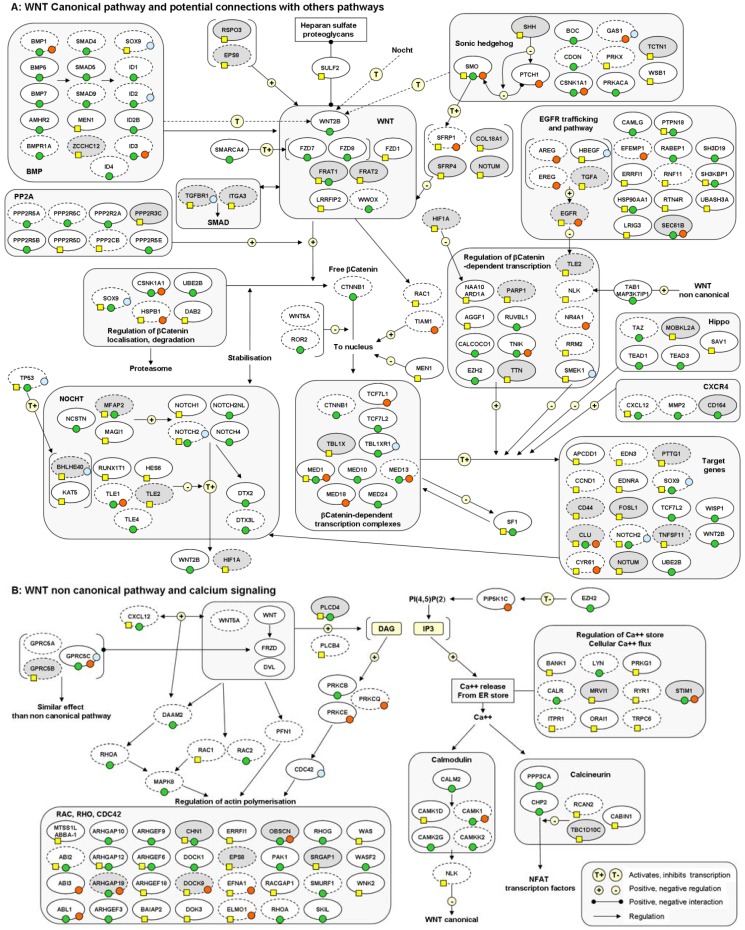
Signal transduction pathways associated with the WNT canonical and noncanonical pathways deregulated in radiation-induced thyroid tumors.The figure gives a simplified overview of the WNT canonical pathway with potential connections with the EGFR, SHH, NOTCH and BMP pathways (**A**) and the WNT noncanonical pathway (**B**). Genes deregulated in post-radiotherapy tumors are indicated by a yellow square, and green or orange circles indicate the genes deregulated in post-Chernobyl tumors, from Ugolin *et al*. [34], or from other studies, respectively. Genes selected in either the post-radiotherapy or the post-Chernobyl signatures (high discriminating potential, see supplementary data) [33,34], are indicated in grey boxes. Blue circles indicate the genes deregulated in studies analyzing the cellular response of thyroid models to radiation exposure. The dotted line indicates the genes reported to be deregulated (mRNA or protein) in studies of sporadic thyroid tumorigenesis.

Comparative analysis of the pathways deregulated in a post-radiotherapy and in a post-Chernobyl series [33,34], with the restriction that the series were not analyzed by using the same arrays, showed, however, that both post-Chernobyl and post-radiotherapy PTCs are deregulated in common molecular pathways associated with cellular response to radiation and oxidative stress, or signal transduction [50]. Particularly, the WNT canonical and noncanonical pathways, and other potentially co-deregulated pathways (Sonic hedgehog, Notch, and EGF pathways), which were first identified in post-radiotherapy tumors [33], were also deregulated in post-Chernobyl tumors (Figure 1). This strongly suggests that post-Chernobyl and post-radiotherapy tumors display a common core of molecular markers, while a fully comparable analysis of post-radiotherapy and post-Chernobyl tumors is needed to decide on the similarities and specificities of thyroid tumors following external exposure or contamination. Moreover, the 5-gene overlap between the 106-gene post-Chernobyl and the 322-gene post-radiotherapy discriminating signatures can classify both series of tumors [50]. Overall, this data suggests that the molecular imprinting found in radiation-induced tumors likely includes the molecular consequences of the initial stress associated with radiation exposure in thyroid tissues and that part of this imprinting is independent of dose, dose rate and route of exposure, at least for doses above 0.1 Gy.

These data are in agreement with the similar risk factors found for thyroid tumors either at Chernobyl or following external radiation exposure. It should also be noted that a common signature was found for both thyroid adenomas and PTC [33], which is also in accordance with epidemiological data that showed similar risk factors for both.

Whether this imprinting denotes exposure to previous radiation or is a hallmark of radiation-tumorigenesis remains to be shown, by comparing gene expression in non tumoral thyroid tissue previously exposed or not to radiation. 

## 7. The Persistent Problem of Low Doses: Can We Evaluate the Risk of Thyroid Cancers for Doses Lower Than 0.1 Gy?

As reported in the studies mentioned above and many others, an increase in thyroid tumor incidence is expected and could be measured by conventional epidemiology for doses above 0.1 Gy, but for lower doses, the risk is not proven. For radiation protection purposes, a linear non-threshold model is applied to extrapolate the risk observed at high doses to low doses [51,52,53]. But strictly, evaluation of such a risk is outside the limits of conventional epidemiology and we do not know to date if a thyroid exposure to such low doses could effectively lead to the development of thyroid tumors.

In any case at low doses, if this risk does exist, a very low number of radiation-induced thyroid tumors is expected and will be masked among the high number of sporadic tumors which has regularly increased all over the world for more than 25 years due to better detection and diagnosis of thyroid diseases [54,55,56,57]. To be used for the detection of these radiation-induced tumors, any test should have predictive values close to 100%.

Because of a lack of identified low-dose radiation induced thyroid tumors, we do not know if the molecular signatures found in tumors that developed after exposure to doses higher than 0.1 Gy are relevant for screening tumors induced by radiation at very low doses. The initial level of stress may lead to molecular variations in the cellular response and to the selection of different molecular pathways/markers in the tumors. Moreover, radiation-induced tumorigenesis may be modulated by specific genetic backgrounds conferring increased radiosensitivity and/or thyroid cancer-prone susceptibility as suggested by Detours *et al*. [29], which could be suspected to have a greater weight at low doses.

## 8. Conclusions and Perspectives

Enough data are now available to show that radiation exposure of the thyroid leads to the deregulation of molecular markers that can be identified and possibly used for etiology prediction, at least for the range of doses for which a significant increase in thyroid tumors can be measured by conventional epidemiology (more than 0.1 Gy). However, all the data presented here should be considered as preliminary and special caution should be taken to avoid specific pitfalls when analyzing molecular specificities of small series of radiation-induced thyroid tumors. All these data should be confirmed using larger series of tumors of good quality in terms of RNA Integrity Number (RIN) index [58], with the same microarray and hybridization techniques for easier comparisons. For example, some studies used tumor-paired normal thyroid tissue for data normalization while others used a pool of normal thyroid tissues as an external reference. The methods used to find the signature should be adapted to small series of samples. This point is important in fully exploring tumor heterogeneity and in limiting the impact of confounding factors. Blind validation of the signatures on independent sets of tumors as large as possible is essential to check the pertinence of the signatures. As some data suggests that thyroid tumors that develop after external exposure (radiotherapy) or ^131^I contamination display a common core of molecular markers, co-analysis of post-radiotherapy and post-Chernobyl tumors may lead to the identification of a “general” radiation-induced signature (for doses higher than 0.1 Gy). This will also help to improve knowledge of radiation-induced tumorigenesis by allowing full and rigorous analysis of the specificities and similarities of the molecular mechanisms selected as a function of the mode of exposure, the dose and the dose rate. Prospective studies will also have to determine what is behind the observed deregulation in radiation-induced tumors: initial stress effect and/or individual susceptibility to radiation exposure and/or thyroid cancer-prone susceptibility. To date, we have no clue to estimate what could be the influence of susceptibility compared with the impact of the initial stress in radiation-induced thyroid tumors.

For the moment the identified signatures cannot be used to screen for tumors induced by very-low-dose radiation, for 2 main reasons: (1) the impact of very-low-dose exposure on thyroid tumor risk is still unproved, and if it does exist it is extremely small and any biological test should have predictive values close to 100% to be used for diagnosis of the few radiation induced thyroid tumors among a much larger number of sporadic ones; (2) if the risk exists, we do not know what the signature is and if it has something in common with high-dose signatures. Radiation exposure at very low doses in animal models that do not develop spontaneous thyroid tumors is needed to estimate the risk. If tumors are observed, comparison of molecular signatures of tumors that develop in mice after very low and high-dose radiation exposure should be made to define molecular markers and similarities and specificities according to doses. 

## Supplementary Files

Supplementary File 1 DOC-Document (DOC, 30 KB)

Supplementary File 2 XLS-Document (XLS, 2373 KB)
