# Supplementary material for: Discriminating Gene Expression Signature of Radiation-Induced Thyroid Tumors after Either External Exposure or Internal Contamination
Source: Genes (Basel). 2011 Dec 21;3(1):19–34. doi: 10.3390/genes3010019 (PMC3899964; doi:10.3390/genes3010019)
Supplement: Supplementary File 1 — DOC-Document (DOC, 30 KB) [file genes-03-00019-s001.doc]

Supplementary data: The EMts-PCA method

The EMts_2PCA method used to identify discriminating signatures of radiation-induced tumors was described in (Ugolin, Ory *et al*. 2011). Briefly, combinatorial matrices (4 radiation-induced tumors *vs.* 4 sporadic tumors) were built from a learning/training set, the remaining tumors of each combinational matrix being used as training tumors. All combinatorial matrices that may introduce a bias in the gene selection due to a known confounding factor (sex, gene alteration...) were discarded. In each combinatorial matrix, a learning step, based on a classification Expectation-Maximization
(EM) algorithm, permitted the selection of a subset of candidate genes whose expression discriminates between the two subgroups. This subset is retained only if it correctly classifies at least one training tumor but never misclassifies any training tumors of each combinatorial matrix, other tumors being unclassified. Then, validated subsets were compiled in a unique discriminating final signature of the most relevant genes (found in at least 70% of validated subsets) for prediction of etiology. This final signature is then validated by the blind classification, case-by-case, of an independent set of tumors (testing tumors). To get an overview of the molecular pathways deregulated in radiation-induced tumors, in addition to the genes included in the final signature, the genes of each validated subset not retained in the final signature are nonetheless considered as putative deregulated genes, since although more heterogeneous among the tumors they were deregulated in some tumors.
